# Trends of the Extra-Hepatic Biliary Cancer and Its Surgical Management: A Cross-Sectional Study From the National Cancer Database

**DOI:** 10.7759/cureus.27584

**Published:** 2022-08-01

**Authors:** Faiz Tuma, Ali Abbaszadeh-Kasbi, Gitonga Munene, Saad Shebrain, William C Durchholz

**Affiliations:** 1 Surgery, Central Michigan University College of Medicine, Saginaw, USA; 2 Surgery, Western Michigan University Homer Stryker MD School of Medicine, Kalamazoo, USA; 3 Surgery, Mayo Clinic, Jacksonville, USA; 4 Surgical Oncology, Western Michigan University Homer Stryker MD School of Medicine, Kalamazoo, USA

**Keywords:** extrahepatic cholangiocarcinoma, surgical management, national cancer database, robotic biliary surgery, extrahepatic biliary cancers

## Abstract

Introduction

Biliary cancers are rare cancers with poor prognoses. In this study, we aimed to evaluate trends in early detection and surgical treatment and approaches in extra-hepatic biliary tract cancers (EBCs) over 13 years in the US.

Methods

The most recent data on patients diagnosed with EBC between 2004 and 2016 were extracted from the National Cancer Database (NCDB). The patients’ demographics (sex, age, race), primary tumor sites, tumor grades and stages, staging modalities, diagnostic confirmation, surgical treatment modalities and approaches, and 90-day mortality were analyzed to determine trends.

Results

Biopsy was the most common staging modality in 63.9% of total 60,291 patients. The bile duct was the primary tumor site (55.0%). Histologic examination was the most common confirmatory diagnostic modality (77.5%). The most common stage was stage II (23%). The most common surgical treatment modality was radical surgery (13.88%). The open surgical approach was used in 27.1% of patients, followed by a laparoscopic approach (4.3%).

Conclusion

EBC showed no significant change in the trends of the stage at diagnosis, treatment modality, and extent of surgical procedures despite advances in surgical diagnostic and therapeutic modalities; however, the total number of cases slightly increased between 2004 and 2016.

## Introduction

Extrahepatic biliary tract cancers (EBCs) are rare, with a reported annual diagnostic rate of 12,000 in the USA [1]. It includes perihilar cholangiocarcinoma, bile duct, gallbladder, and distal or ampullary cancers. Compared to intrahepatic biliary tract cancers, they are more common and tend to cause symptoms more often. However, clinical differentiation of the types is unreliable since most of these cancers will cause mass-like lesions and lead to obstruction of the biliary tract. Surgical treatment, whether radical or limited/palliative, varies according to the location [2, 3].

Over the last two decades, there have been shifts in the diagnostic and treatment modalities for cancer. The effects of new diagnostic tools and treatment approaches are reflected in the outcome objectives of treatment, which favor early/accurate detection and less aggressive procedures, including robotic and laparoscopic surgery. Early detection potentially leads to less radical procedures and better outcomes. Implementation of minimally invasive surgery for biliary cancers at the early stages, as for several other cancers, has increased over the years for several reasons [4, 5]. Studies have shown that minimally invasive approaches are associated with less postoperative pain, shorter length of stay, and faster recovery time [6].

Diagnosing biliary cancers at an early stage will potentially increase survival rates by enabling less aggressive surgical treatment for localized diseases. In addition, the continuously improving surgical technology available for diagnostic workup and intervention, including imaging modalities and endoscopy with tissue acquisition capabilities, may enhance earlier detection. Therefore, the objective of this study was to evaluate EBC trends over 13 years, including early detection, the extent of surgical treatment, and the surgical approaches.

## Materials and methods

Data source

Data on patients with biliary cancer between 2004 and 2016 were extracted from the most recently available National Cancer Database (NCDB), a nationally recognized database sponsored by the American College of Surgeons and the American Cancer Society [6]. The NCDB collects data on approximately 70% of patients [7] newly diagnosed with cancer from more than 1500 Commission on Cancer-accredited cancer facilities throughout the US. This de-identified database is not verified by the sponsoring organizations, so they are not responsible for the validity of the data or the conclusions drawn by the authors. The database is accredited and approved by the American College of Surgeons and American Cancer Society and is in accordance with the Declaration of Helsinki. The data of patients with biliary tract cancer with primary cancer sites of the extrahepatic duct, the ampulla of Vater, overlapping sites of the biliary tract, or unspecified primary sites were reviewed. The study was deemed exempt by the Institutional Review Board at our institution.

Statistical analysis

Descriptive statistics were analyzed, with continuous data reported as mean (SD) and categorical data reported as frequency (percent). We used ANOVA with posthoc analysis to evaluate the differences between ages across years of study. The difference in proportions was assessed by Pearson’s chi-squared test. Posthoc analysis involved pairwise comparisons with a Bonferroni correction. The variables analyzed in this study were the patients’ demographics (sex, age, and race) and clinical variables, including diagnostic and staging modalities, diagnostic confirmation modalities, surgical treatment approaches, primary tumor sites, tumor grading, tumor staging, year of diagnosis, and surgical treatment modalities. Tumor grades are presented as follows: grade I, well-differentiated; grade II, moderately differentiated; grade III, poorly differentiated; grade IV, undifferentiated, and cell type not determined (not stated, not applicable, or high-grade dysplasia [adenocarcinoma in situ]). Tumor stages are presented according to the American Joint Committee on Cancer (AJCC) staging system. Data analysis was completed using SPSS version 26 software (IBM Corp, Armonk, NY). Statistical significance was established at p <0.05.

## Results

A total of 60,291 cases of biliary cancer between 2004 and 2016 from the NCDB were reviewed. Of those cases, 32,522 (53.9%) patients were men, while 27,769 (46.1%) patients were female, with a male-to-female ratio of 1.17:1. The most common race was white (83.4%), followed by black (8.9%). There was a slightly statistically significant difference in patients' sex, as assessed by Pearson’s chi-squared test (p = 0.013). Posthoc analysis involved pairwise comparisons with a Bonferroni correction. Statistical significance was accepted at p <0.0038. Overall, the mean (SD) age of the entire study population was 70.2 (12.5) years (Table 1). One-way ANOVA was conducted and showed statistically significant differences in mean age across groups (p=0.002). However, posthoc analysis only showed a statistically significant mean difference between the years 2008 and 2012 (p=0.032). A difference was not found between other years. Overall, there was a slight and progressive increase in the recorded cases of EBC over the 13 years. This trend was not statistically significant regarding extrahepatic bile duct cancer when pairwise comparisons with a Bonferroni correction were performed (Table 1, footnote). However, there were statistically significant differences among other tumors with variability yearly.

**Table 1:** Demographics: age and primary site of tumor. *Each subscript letter denotes a subset of years (2004 through 2016) categories whose column proportions do not differ significantly from each other at the 0.05 level (Pearson's Chi-squared, 71.509 df = 36, p <0.001).

	2004	2005	2006	2007	2008	2009	2010	2011	2012	2013	2014	2015	2016	Total
A. Mean (SD) at diagnosis (years)	70.8 (12.5)	70.5 (12.8)	70.6 (12.7)	70.4 (12.6)	71.0 (12.6)	70.1 (12.8)	69.8 (12.5)	69.8 (12.8)	69.7 (12.4)	70.1 (12.5)	69.8 (12.3)	70.2 (12.1)	70.3 (11.7)	70.2 (12.5)
B. Primary site, n (%)														
Extrahepatic bile duct*	1941_a_ (53.57)	2060_a_ (55.63)	2139_a_ (54.11)	2172_a_ (54.37)	2333_a_ (54.83)	2560_a_ (56.93)	2659_a_ (56.49)	2788_a_ (56.57)	2777_a_ (55.32)	2939_a_ (54.37)	2962_a_ (55.08)	2997_a_ (54.68)	2866_a_ (53.62)	33193
Malignant neoplasm of ampulla of Vater*	1340_a_ (36.99)	1260_a,b,c_ (34.03)	1394_a,c_ (35.26)	1368_a,b,c_ (34.24)	1445_a,b,c_ (33.96)	1435_b_ (31.91)	1529_b,c_ (32.48)	1600_b,c_ (32.47)	1672_a,b,c_ (33.31)	1840_a,b,c_ (34.04)	1780_b,c_ (33.10)	1848_a,b,c_ (33.72)	1873_a,b,c_ (35.04)	20384
Malignant neoplasm of overlapping sites of biliary tract*	36_a_ (0.99)	37_a_ (1.00)	49_a_ (1.24)	46_a_ (1.15)	46_a_ (1.08)	52_a_ (1.16)	42_a_ (0.89)	38_a_ (0.77)	50_a_ (1.00)	60_a_ (1.11)	51_a_ (0.95)	58_a_ (1.06)	43_a_ (0.8)	608
Malignant neoplasm of biliary tract, unspecified*	306_a_ (8.45)	346_a,b_ (9.34)	371_a,b_ (9.39)	409_a,b_ (10.24)	431_a,b_ (10.13)	450_a,b_ (10.01)	477_a,b_ (10.13)	502_a,b_ (10.19)	521_a,b_ (10.38)	567_b_ (10.49)	585_b_ (10.88)	578_a,b_ (10.55)	563_b_ (10.53)	6106
Total	3623	3703	3953	3995	4255	4497	4707	4928	5020	5406	5378	5481	5345	60291

Diagnostic and staging procedures

A biopsy (including incisional, needle, or aspiration) of the primary site or other sites (metastasis) was the most commonly used procedure for diagnostic and staging (N = 38526, 63.9%). Conversely, no surgical procedure for staging was performed in 20,192 (33.4%) patients (Table 2).

**Table 2:** Diagnostic and staging procedures, and confirmation modules. ₡ Biopsy (incisional, needle, aspiration) was done to either primary or another site. ₵ Include surgical exploration without biopsy, unknown type of procedure, or no information of whether a diagnostic or staging procedure was performed. ¥ Tissue microscopically examined. #The malignancy was reported by the physician from an imaging technique report only. ₸ No tissue microscopically examined; only fluid cells microscopically examined. *The tumor was visualized during a surgical/endoscopic procedure only with no tissue resected for microscopic examination. ** Other modalities are laboratory marker (a clinical diagnosis of cancer is made according to the laboratory tests/marker studies), microscopic examination (It is unknown if the cells were from cytology or histology), histology plus immunophenotyping and/or genetic studies, or a statement of malignancy was reported in the medical record, but there is no statement how the cancer was diagnosed. ‡The malignancy was reported by physician in medical record.

	2004	2005	2006	2007	2008	2009	2010	2011	2012	2013	2014	2015	2016	Total
Diagnostic and staging procedures, n (%)														
Tissue Biopsy₡	2163 (59.7)	2196 (59.3)	2320 (58.6)	2338 (58.5)	2606 (61.2)	2778 (61.7)	3018 (64.1)	3239 (65.7)	3333 (66.4)	3537 (65.4)	3623 (67.3)	3725 (67.9)	3650 (68.2)	38526 (63.9)
Surgical procedure with bypass and without tissue biopsy	61 (1.6)	69 (1.8)	57 (1.4)	63 (1.5)	58 (1.3)	68 (1.5)	62 (1.3)	59 (1.1)	47 (0.9)	43 (0.7)	31 (0.5)	24 (0.4)	27 (0.5)	669 (1.1)
Other procedures₵	73 (2.0)	108 (2.9)	94 (2.3)	85 (2.1)	78 (1.8)	87 (1.9)	61 (1.3)	47 (0.9)	66 (1.3)	56 (1.0)	50 (0.9)	54 (0.9)	45 (0.8)	904 (1.5)
No surgical procedure	1326 (36.5)	1330 (35.9)	1482 (37.4)	1509 (37.7)	1513 (35.5)	1564 (34.7)	1566 (33.2)	1583 (32.1)	1574 (31.3)	1770 (32.7)	1674 (31.1)	1678 (30.6)	1623 (30.3)	20192 (33.4)
Diagnostic confirmation modules, n (%)														
Histology ¥	2713 (74.8)	2720 (73.4)	2904 (73.4)	2989 (74.8)	3222 (75.7)	3431 (76.2)	3649 (77.5)	3823 (77.5)	3955 (78.7)	4246 (78.5)	4309 (80.1)	4396 (80.2)	4372 (81.7)	46729 (77.5)
Radiology and/or imaging techniques without microscopic examination #	154 (4.2)	163 (4.4)	196 (4.9)	201 (5.0)	193 (4.5)	181 (4.0)	160 (3.3)	187 (3.7)	194 (3.8)	195 (3.6)	173 (3.2)	179 (3.2)	147 (2.7)	2323 (3.8)
Cytology₸	579 (15.9)	622 (16.7)	672 (16.9)	626 (15.6)	662 (15.5)	705 (15.6)	737 15.6%	760 15.4%	706 14.0%	793 14.6%	748 13.9%	772 14.0%	705 13.1%	9087 15.0%
Direct visualization without microscopic confirmation*	100 (2.7)	99 (2.6)	74 (1.8)	71 (1.7)	70 (1.6)	71 (1.5)	55 (1.1)	51 (1.0)	48 (0.9)	55 (1.0)	33 (0.6)	24 (0.4)	30 (0.5)	781 (1.3)
Other modalities**	23 (0.6)	31 (0.8)	32 (0.8)	35 (0.8)	42 (0.9)	37 (0.8)	37 (0.7)	39 (0.8)	42 (0.8)	33 (0.6)	40 (0.7)	42 (0.7)	28 (0.5)	451 (0.7)
Clinical diagnosis only‡	54 (1.5)	68 (1.8)	75 (1.9)	73 (1.8)	66 (1.5)	72 (1.6)	69 (1.4)	68 (1.3)	75 (1.5)	84 (1.5)	75 (1.4)	78 (1.4)	63 (1.1)	920 (1.5)

Diagnostic confirmation modalities

Histologic examination was the most common diagnostic confirmation modality (N = 46,729, 77.5%) and cytology was the second most common modality used (N = 9,087, 15%), followed by radiographic and imaging techniques (N = 2,323, 3.8%) (Table 2).

Primary tumor site

**Figure 1:**
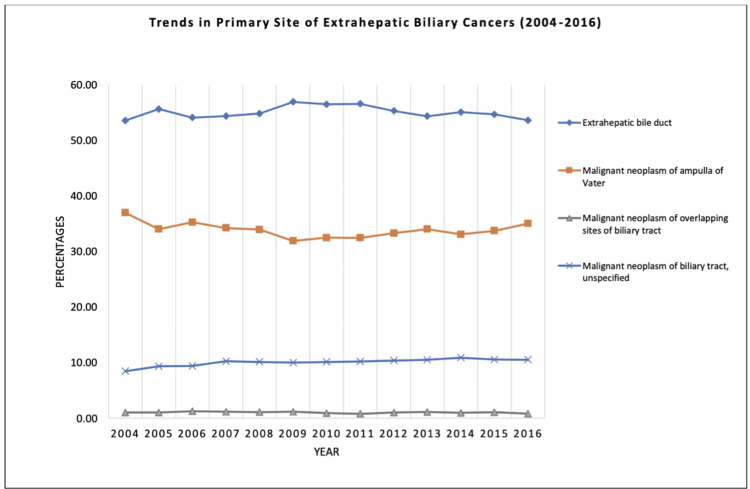
Trends in primary site of extrahepatic biliary cancers between 2004 and 2016.

**Figure 2:**
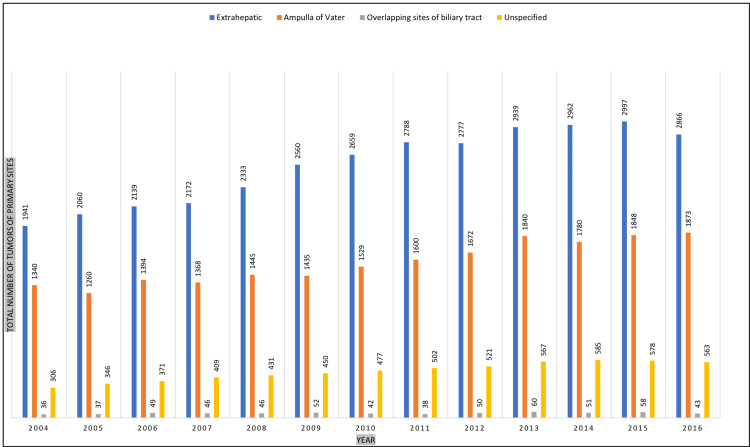
Demonstrating the total number of tumors pf primary sites from 2004 to 2016.

The bile duct, the ampulla of Vater, and overlapping biliary tract sites were the primary tumor sites in 33,193 (55.0%), 20,384 (33.8%), and 608 (1.0%) patients, respectively. However, the primary tumor site was unspecified in 6,106 (10.1%) patients (Table 1 and Figures 1-2).

Grading and staging

Tumors were well-differentiated (Grade I), moderately differentiated (Grade II), poorly differentiated (Grade III), undifferentiated (Grade IV), and undetermined in 4,183 (6.9%), 14,289 (23.7%), 11,250 (18.6%), 462 (0.7%), and 30,107 (49.9%) patients, respectively. According to the AJCC staging system, 764 (1.2%), 10645 (17.6%), 13,918 (23%), 6674 (11%), and 11813 (19.6%) patients had cancer stages 0, I, II, III, and IV, respectively (Table 3).

**Table 3:** Tumor grade and AJCC stage per year. ₦ Cell type not determined, not stated or not applicable; unknown primaries; high-grade dysplasia (adenocarcinoma in situ).

	2004	2005	2006	2007	2008	2009	2010	2011	2012	2013	2014	2015	2016	Total
Tumor grade, n (%)														
Well differentiated	304 (8.4)	293 (7.9)	242 (6.1)	268 (6.7)	274 (6.4)	296 (6.5)	339 (7.2)	374 (7.5)	326 (6.5)	406 (7.5)	365 (6.7)	358 (6.5)	338 (6.3)	4183 (6.9)
Moderately differentiated	830 (22.9)	847 (22.8)	998 (25.2)	932 (23.3)	987 (23.2)	1045 (23.2)	1088 (23.1)	1122 (22.7)	1199 (23.8)	1261 (23.3)	1287 (23.9)	1336 (24.3)	1357 (25.3)	14289 (23.7)
Poorly differentiated	699 (19.2)	702 (18.9)	719 (18.1)	693 (17.3)	838 (19.6)	854 (19.0)	857 (18.2)	939 (19.0)	932 (18.5)	998 (18.1)	1033 (19.2)	982 (17.9)	1004 (18.7)	11250 (18.6)
Undifferentiated	34 (0.9)	31 (0.8)	23 (0.5)	28 (0.7)	39 (0.9)	36 (0.8)	28 (0.6)	41 (0.8)	27 (0.5)	41 (0.7)	39 (0.7)	44 (0.8)	51 (0.9)	462 (0.7)
Undetermined₦	1756 (48.4)	1830 (49.4)	1971 (49.8)	2074 (51.9)	2117 (49.1)	2266 (50.3)	2395 (50.8)	2452 (49.7)	2536 (50.5)	2700 (49.9)	2654 (49.3)	2761 (50.3)	2595 (48.5)	30107 (49.9)
AJCC Stage per year, n (%)														
0	54 (1.5)	54 (1.4)	52 (1.3)	53 (1.3)	44 (1.0)	67 (1.4)	67 (1.4)	55 (1.1)	54 (1.0)	73 (1.3)	50 (0.9)	85 (1.5)	56 (1.0)	764 (1.2)
I	669 (18.4)	634 (17.1)	749 (18.9)	719 (18.0)	809 (19.0)	917 (20.4)	805 (17.1)	843 (17.1)	846 (16.8)	905 (16.7)	899 (16.7)	970 (17.6)	880 (16.4)	10645 (17.6)
II	847 (23.3)	838 (22.6)	881 (22.2)	868 (21.7)	1020 (23.9)	1080 (24.0)	1036 (22.0)	1048 (21.2)	1129 (22.5)	1262 (23.3)	1309 (24.3)	1293 (23.5)	1307 (24.4)	13918 (23.0)
III	340 (9.3)	386 10.4%	383 9.6%	403 10.0%	453 10.6%	481 10.6%	516 10.9%	613 12.4%	621 12.3%	634 11.7%	631 11.7%	609 11.1%	604 11.3%	6674 11.0%
IV	607 (16.7)	650 (17.5)	704 (17.8)	694 (17.3)	882 (20.7)	928 (20.6)	922 (19.5)	968 (19.6)	970 (19.3)	1116 (20.6)	1118 (20.7)	1139 (20.7)	1115 (20.8)	11813 (19.6)
Staging not applicable	0 (0)	3 (0.08)	6 (0.1)	4 (0.1)	8 (0.1)	2 (0.04)	533 (11.3)	556 (11.2)	589 (11.7)	646 (11.9)	657 (12.2)	654 (11.9)	637 (11.9)	4295 (7.1)
Staging unknown, n (%)	1106 (30.5)	1138 (30.7)	1178 (29.8)	1254 (31.3)	1039 (24.4)	1022 (22.7)	828 (17.6)	845 (17.1)	811 (16.1)	770 (14.2)	714 (13.2)	731 (13.3)	746 (13.9)	12182 (20.2)

Surgical approach

**Figure 3:**
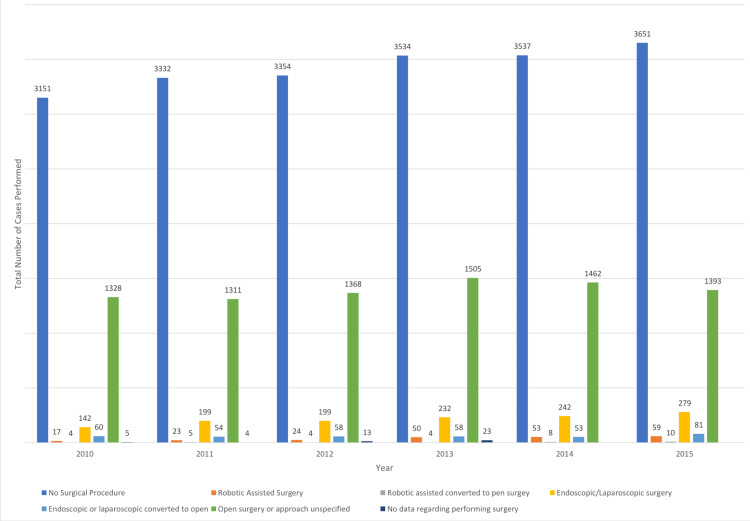
Surgical approaches per year.

An open surgical approach was used in 9837 (27.1%) patients followed by laparoscopic (N = 1575, 4.3%) and robotic (N = 309, 0.8%) approaches (Table 4, Figure 3). The NCDB began tracking data on surgical approaches in 2010, so complete data is only available from 2010 to 2016. The use of the robotic-assisted approach has been steadily increasing over the seven years of the study to five-fold. The laparoscopic approach increased two-fold. In comparison, the open approach decreased only about 10% over the same period. 

**Table 4:** Surgical approaches.

Surgical approaches	2010	2011	2012	2013	2014	2015	2016	Total
No surgical procedure of primary site at this facility, n	3151	3332	3354	3534	3537	3651	3438	23997
Robotic assisted, n	17	23	24	50	53	59	83	309
Robotic converted to open, n	4	5	4	4	8	10	9	44
Endoscopic or laparoscopic, n	142	199	199	232	242	279	282	1575
Endoscopic or laparoscopic converted to open, n	60	54	58	58	53	81	58	422
Open or approach unspecified, n	1328	1311	1368	1505	1462	1393	1470	9837
Unknown whether surgery was performed at this facility, n	5	4	13	23	23	8	5	81
Total, N	4707	4928	5020	5406	5378	5481	5345	36265

Surgical treatment modalities

Radical surgery (N = 8,369, 13.88%) and total local tumor removal (N = 7,199, 11.94%) were the most common surgical treatments. Other modalities include electrocautery (fulguration modality), local tumor excision (not otherwise specified [NOS]), local tumor destruction (NOS), laser, laser ablation, laser excision, photodynamic therapy, cryosurgery, polypectomy, excisional biopsy, simple/partial surgical removal of the primary site, debulking surgery, and surgery (NOS) were used much less frequently (Table 5). No surgical treatment was performed in 32,267 (60.1%) patients. Given the multiple comparisons with Bonferroni adjustment, surgical treatment extent and modality showed no significant trend change for all modalities at p >0.0006.

**Table 5:** Surgical treatment extent and modalities used to treat biliary cancer per each year from 2004 to 2016. * Include local tumor destruction (NOS), local tumor excision (NOS), electrocautery, photodynamic therapy (PDT), cryosurgery, laser, laser ablation, laser excision, polypectomy, excisional biopsy, debulking surgery, and unknown whether surgery was performed.

	2004	2005	2006	2007	2008	2009	2010	2011	2012	2013	2014	2015	2016	Total
No surgical treatment, n (%)	2142 (59.12)	2268 (61.24)	2425 (61.34)	2414 (60.42)	2622 (61.62)	2752 (61.19)	2873 (61.03)	3017 (61.22)	3058 (60.91)	3209 (59.35)	3178 (59.09)	3262 (59.51)	3047 (57.0)	36267 (60.1)
Simple/Partial surgical removal of primary site, n (%)	303 (8.36)	276 (7.45)	300 (7.58)	323 (8.08)	380 (8.93)	380 (8.45)	448 (9.51)	472 (9.57)	490 (9.76)	539 (9.97)	535 (9.94)	523 (9.54)	528 (9.87)	5497 (9.11)
Total surgical removal of primary site (enucleation), n (%)	424 (11.70)	420 (11.34)	435 (11.00)	430 (10.76)	436 (10.24)	528 (11.74)	545 (11.57)	560 (11.36)	576 (11.47)	685 (12.67)	708 (13.16)	703 (12.82)	749 (14.01)	7199 (11.94)
Radical surgery, n (%)	548 (15.12)	530 (14.31)	603 (15.25)	588 (14.71)	603 (14.17)	592 (13.16)	605 (12.85)	636 (12.90)	663 (13.20)	737 (13.63)	739 (13.74)	756 (13.79)	769 (14.38)	8369 (13.88)
Surgery (NOS), n (%)	116 (3.20)	94 (2.53)	82 (2.07)	91 (2.27)	94 (2.20)	103 (2.29)	108 (2.29)	118 (2.39)	99 (1.97)	102 (1.88)	88 (1.63)	74 (1.35)	96 (1.79)	1265 (2.09)
Other modalities*, n (%)	90 (2.4)	115 (3.1)	108 (2.7)	149 (3.7)	120 (2.8)	142 (3.1)	128 (2.7)	125 (2.5)	134 (2.6)	134 (2.4)	130 (2.4)	163 (2.9)	156 (2.9)	1694 (2.8)

Ninety-day mortality

Ninety-day mortality was reviewed (Table 6). The available data included the years 2004 to 2015, as the NCDB does not contain data for 90-day mortality for the year 2016. There was a slight decrease in the 90-day mortality from 2004 (10%) to 2015 (8.3%). Therefore, the 90-day mortality was compared across the 12 years. Overall, there was a slight statistically significant difference in proportions, as assessed by Pearson’s Chi-square, p = 0.047. Posthoc analysis involved pairwise comparisons with a Bonferroni correction. Statistical significance was accepted at p < 0.00041. Therefore, 90-day mortality was not statistically significant across years (2004-2015) as assessed by Pearson’s Chi-square, p > 0.00041. NCDB data does not provide long-term mortality information.

**Table 6:** 90-day mortality per each year from 2004 to 2015.

	2004	2005	2006	2007	2008	2009	2010	2011	2012	2013	2014	2015	Total
Alive by 90 days, n (%)	1309 (91.0)	1263 (90.4)	1347 (90.8)	1388 (91.2)	1468 (92.3)	1550 (91.6)	1644 (92.6)	1719 (92.8)	1739 (92.8)	1928 (91.3)	1959 (93.2)	1899 (92.3)	19213 (91.9)
Dead by 90 days, n (%)	130 (9.0)	134 (9.6)	136 (9.2)	134 (8.8)	122 (7.7)	142 (8.8)	132 (7.4)	133 (7.2)	142 (7.5)	184 (8.7)	143 (6.8)	159 (7.7)	1691 (8.1)

## Discussion

Biliary cancer is a rare disease in North America; it is more common in Asia due to parasitic diseases that can cause bile duct cancers [8]. Compared to the unchanged trends of all cancers and liver cancers [9], the EBC trend is similar in that it has not changed significantly. The demographics of EBCs did not show a significant difference over the 14 years studied. The maximum age of diagnosis was 77 years; however, it can occur at any age [8]. There was an almost equal male-to-female ratio of 1.17:1. The most common race was white (83.4%), followed by black (8.9%). This is different from the general racial distribution of cancers in general, where non-Hispanic blacks have higher incidence rates [9].

Diagnostic confirmation

Histologic examination was the most common confirmatory diagnostic modality (77.5%), followed by a much lower rate of radiologic imaging. Different imaging modalities are available for diagnosis and staging, such as ultrasound (contrast-enhanced, intraductal, and endoscopic), CT, positron emission tomography (PET), MRI, and direct cholangiography [10]. Endoscopic ultrasonography (EUS) is increasingly recognized as an important diagnostic tool for pancreaticobiliary malignancies that evaluates duct morphology, regional lymphadenopathy, and vascular involvement and facilitates tissue acquisition [11]. EUS-guided fine-needle tissue acquisition may establish the biliary obstruction diagnosis with a sensitivity ranging from 27% to 83% for malignancy [12]. The range is wide and will likely improve with improved techniques and expertise.

Grade

The most common grade among the determined cases was moderately differentiated (Grade II), followed by poorly differentiated (Grade III). There was no trend change from 2004 to 2016. Biliary tract cancers are known to be biologically aggressive with poor prognoses [1]. Fifty percent of the reviewed cases were undetermined for unknown reasons. This large percentage of patients could statistically determine the overall behavior of cancer. Three histological types of cholangiocarcinoma that influence the grade and cancer behavior have been identified: papillary, tubular, and superficial spreading types [13, 14]. Histological grade III cancers and rare variants with squamous and sarcomatous differentiation are associated with poor prognoses, while mucinous carcinoma has a better prognosis [15].

Stage at diagnosis

EBCs are commonly diagnosed at an advanced stage with limited treatment options and poor prognoses [16, 17]. They are less likely to be diagnosed at earlier stages due to the rarity of the disease, lack of screening and simple diagnostic tests, and the asymptomatic nature of the early stages. Hence, most of these cancers are diagnosed when symptomatic. Mass effects and obstruction leading to jaundice are the most common presenting symptoms. Occasionally, incidental cases are identified while investigating other diseases. Therefore, biliary cancer is usually diagnosed in the late stages of the disease. There was no noticeable change in the trends of the stage at diagnosis from 2004 to 2016. The stages from II to IV were almost evenly distributed, comprising between 16% and 21% each. The stage at diagnosis was expected to change over 12 years with the availability of advanced diagnostic imaging technology and endoscopic skills through EUS, which provides more information on suspected cases using the less aggressive intervention. However, the other cancer characteristics, such as late clinical presentation, hidden location, and the absence of screening, seem to have been a more important influence on the stage at diagnosis.

Surgical treatment

While most biliary cancer patients present when the disease is unresectable, the treatment of choice for early biliary cancers is surgical resection, even though the risk of recurrence is high [18]. Localized diseases are generally considered resectable, whereas locally advanced or distant metastatic diseases are beyond the scope of surgery, and resection of nodal metastatic disease is still controversial [15]. Patients with biliary cancer should be offered surgical resection as the only treatment associated with long-term survival and potential cure since there has been little progress in the development of locoregional treatments [15]. Surgical treatment extent and modality showed no significant trend change for all modalities and approaches. The most common modality was radical surgery (around 14% of total procedures; N = 8,369 of 60,291 surgically treated patients). Radical surgery for perihilar bile duct cancer involves combined hepatic and hilar resection. Previously, hilar resection with limited hepatectomy was performed but with unsatisfactory long-term survival due to positive margins [15]. More recently, extended procedures, including hemihepatectomy or extended hepatectomy, extrahepatic bile duct resection, and regional lymphadenectomy, have been performed due to a better understanding of tumor pathology [19]. In addition, distal cholangiocarcinomas of the distal bile duct are treated with pancreaticoduodenectomy [20, 21].

Surgical approach

The surgical approaches, from completely open to laparoscopic or robotic approaches, demonstrated some changing trends. There was a noticeable increase in the minimally invasive approach in biliary cancer procedures. However, the rate of increase varied between the laparoscopic and the robotic-assisted approaches. The robotic approach increased five-fold from 2010 to 2016 (17 procedures in 2010 vs. 83 procedures in 2016). The absolute number is still small compared to the number of total procedures; however, if it continues at the same rate and direction, the trajectory of increase will be noticeable. Robotic surgery as a minimally invasive approach has been gaining increasing favor due to its many advantages. Its range of applications in various procedures has been quickly expanding, as it has the potential to overcome some of the limits of laparoscopy [5]. Even though the number of procedures performed laparoscopically was more than those performed robotically over the years, the laparoscopic approach did not show the same rate of increase (142 in 2010 vs. 282 in 2016). Given the complexity of biliary procedures, the advanced disease stage at diagnosis, and the rarity of the disease, the prevalence of the minimally invasive approach in biliary cancer may not progress as in other abdominal or GI procedures. This study has limitations. Like all large database studies, data-coding errors could potentially result in misclassification bias and altering the observed association or outcome of interest. Fortunately, there are a large number of patients in this study, which should offset these errors. Additionally, while the NCDB, as a hospital-based, is a powerful resource for quality improvement, generalizability from this data is limited. Furthermore, other limitations include the lack of longitudinal treatment data, clinically relevant endpoints such as morbidity and mortality, patient-reported outcomes, local disease control, and disease-free survival.

## Conclusions

EBCs showed no significant change in the trends of the stage at diagnosis, treatment modality, and extent of surgical procedures, even with the advancement of diagnostic techniques. This is likely due to the rarity of the disease, the asymptomatic nature of the early stages, and possibly the limitations of the available data. However, the surgical approaches showed a noticeable increase in the minimally invasive approach, especially the robotic-assisted approach. Multicentric studies with complete case data and follow-up are needed to further study the disease’s current status.
